# Micronutrients in Adverse Pregnancy Outcomes

**DOI:** 10.12688/f1000research.124960.1

**Published:** 2022-11-23

**Authors:** Sairoz, Krishnananda Prabhu, Ranita Ghosh Dastidar, Annayya Rao Aroor, Mahadev Rao, Sahana shetty, Vidyashree G Poojari, Varashree BS

**Affiliations:** 1 Biochemistry, Kasturba Medical College, Manipal, Manipal Academy of Higher Education, Manipal, Karnataka, 576104, India; 2 Division of Endocrinology and Metabolism, Department of Medicine, University of Missouri, Columbia, Columbia, MO, USA; 3 Pharmacy Practice, Manipal College of Pharmaceutical Sciences, Manipal, Manipal Academy of Higher Education, Manipal, Karnataka, 576104, India; 4 Endocrinology, Kasturba Medical College, Manipal, Manipal Academy of Higher Education, Manipal, Karnataka, 576104, India; 5 Reproductive Medicine and Surgery, Kasturba Medical College, Manipal, Manipal Academy of Higher Education, Manipal, Karnataka, 576104, India

**Keywords:** Micronutrients, Pregnancy, Foetal growth restriction, Gestational diabetes, Iron, spontaneous abortion, zinc, magnesium, copper

## Abstract

About 10 to 20% of reported pregnancies have complications like spontaneous abortion (SA), preeclampsia (PE), preterm birth (PTB), and fetal growth restriction (FGR); 60% are attributed to maternal nutritional alterations. Multiple micronutrients (MMN) are supplemented in the antenatal period, but no proper validation/guidelines are available regarding dosing/time, the need for initiation, and the duration of supplementation. Studies have reported adverse pregnancy complications related to the overuse/unwanted use of multiple micronutrient supplementations during pregnancy. Identifying the exact population requiring supplementation is necessary to prevent its abuse. This article attempts to review the impacts of micronutrient deficiency/supplementation in cases of SA, FGR, and gestational diabetes mellitus (GDM), preterm delivery and PE. The study used a literature search using PubMed, Google Scholar, Mendeley, and Scopus Databases using search words pregnancy, spontaneous abortion, gestational diabetes mellitus (GDM), fetal growth restriction (FGR), preterm delivery, preeclampsia (PE) or “adverse pregnancy” associated with minerals, micronutrients, or supplementation. The review also considered in-house literature databases, a single-window search at Kasturba Medical College (KMC) Health sciences library, MAHE (Manipal Academy of Higher Education). The figures included in the study were created by Biorender.com. Micronutrients play multiple roles during pregnancy and fetoplacental growth stimulating growth hormone secretion, Lysyl oxidase (LOX), involved in the crosslinking between collagen and elastin in the amniotic membrane, downregulation of interleukin (IL)-1 alpha, IL-1 beta, IL-4, IL-6, Il-10, IL-12, tumor necrosis factor (TNF)-alpha and several chemokines involved in hypertension, immune-inflammatory pathways, attenuate insulin resistance a structural development of neurons and glia. Over-supplementation has led to complications such as spontaneous abortion and gestational diabetes mellitus. Since there is a lack of standardization concerning micronutrient supplementation during pregnancy, there is a need for systematic study related to the role of micronutrients during each trimester of pregnancy to optimize its supplementation and to prevent hazards associated with its abuse.

## Introduction

Adverse pregnancy outcomes, pregnancy other than normal livebirths, which majorly include spontaneous abortion (SA), preterm birth (PTB), fetal growth restriction (FGR), and preeclampsia (PE). 10—20% of reported pregnancies have complications like SA, PE, PTB, and FGR. Of these, 60% are attributed to maternal nutritional alterations and 40% to the alteration in the placenta and the fetus. ^1^ The reported adverse outcomes are 19.8% for preterm, 1.7% for spontaneous abortions, 1% for gestational diabetes, and 0.9% for stillbirths in rural India. Inadequate diet/nutrition during pregnancy commonly causes impaired fetoplacental growth and placental functions. ^2^^,^^3^ As trace elements are responsible for the maintenance of cellular structures and processes, any imbalance of micronutrients such as zinc, copper, iron, and magnesium can predispose to problems in conceiving, structural or functional abnormalities in the fetus, fetal death, premature ruptured membranes, and small for gestational age (SGA) babies. ^4^^,^^5^

Based on the above observation, multiple micronutrient supplementation (MMN) is often given during antenatal period. But there are no proper validation/guidelines available concerning dosing/time, the need for initiation and duration of supplementation, etc., which has led to many unwanted supplementations. Studies report adverse pregnancy complications related to the overuse/unwanted use of multiple micronutrient supplementations during pregnancy. ^6^^–^^8^ Identifying the exact population requiring supplementation is necessary to prevent its abuse. This article attempts to review the impacts of micronutrient deficiency/supplementation in cases of spontaneous abortion (SA), fetal growth restriction (FGR), preterm delivery, gestational diabetes mellitus (GDM), and preeclampsia (PE).

## Methods

The study used a literature search using PubMed, Google Scholar, Mendeley, and Scopus Databases using search words “pregnancy”, OR “spontaneous abortion” OR “gestational diabetes mellitus” (GDM) OR “fetal growth restriction” (FGR) OR “preterm delivery” OR “preeclampsia” (PE) OR “adverse pregnancy” associated with “minerals,” “micronutrients,” OR “supplementation.” The review also considered in-house literature databases, a single-window search at Kasturba Medical College (KMC) Health sciences library, MAHE (Manipal Academy of Higher Education). As mentioned above, we identified 5260 articles with the search criteria. The study specifically screened 128 high impact journals such as The American Journal of the Medical Science, Journal of Trace Elements in Medicine and Biology, Nutrients, etc. and included 93 research articles. The review evaluated observational studies and randomized controlled trials since1/1/2015. Meta-analyses: reviews were excluded from this study. For clinical and global importance, the review chose the referred studies.

## Metabolic functions of micronutrients during pregnancy

### Zinc

**Figure 1.**
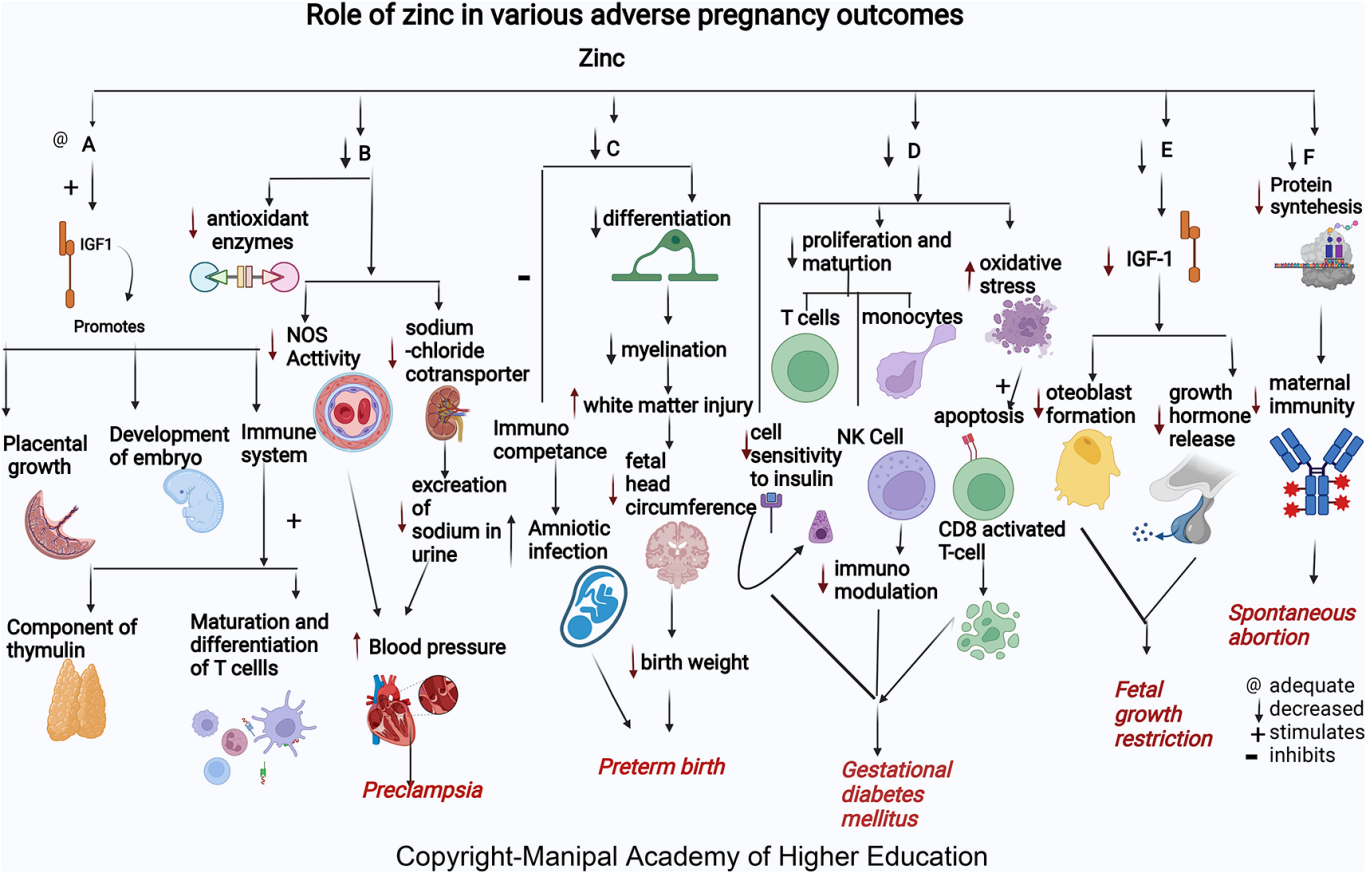
A-Zinc stimulates Insulin-like growth factor1 (IGF1) promotes placental growth, development of embryo and involved in immune system as component of thymulin, maturation and differentiation of T cells. B-Low zinc levels increases blood pressure by reducing the antioxidant enzymes, decreasing the nitric oxide synthase (NOS) activity in the artery and decreased effect on sodium chloride cotransporter in kidney thus reduces excretion of sodium in urine leading Preeclampsia. C-Zinc deficiency causes decreased differentiation of oligodendrocytes, decreased myelination, increased cerebral white matter injury (WMI), and decreased head circumference of the foetus causes early delivery. Lower maternal serum zinc concentration may inhibit immunological competence in bothmother and foetus and therefore increase the risk of amniotic infection and lead to preterm birth. D-Zinc deficiency decreases insulin activity by decreasing cell sensitivity to insulin, decreases proliferation and maturation of T cell, NK Cell and monocytes, increases oxidative stress which induces the apoptotic deletion in the CD8+CD25+activated regulatory T cell, decreases immunomodulation and, hence leads gestational diabetes mellitus. E-Zinc deficiency decreases growth hormones and osteoblast formation via IGF-I in foetus causing foetal growth restriction. F-Zinc deficiency will decrease the protein synthesis thereby decreases the maternal immunity leading spontaneous abortion.

Zinc acts as a cofactor for more than 300 enzymes, involved in the regulation of cell growth, hormone release, organ development (kidney and heart), immunological response, maturation, and differentiation of T cells, and reproduction (ovarian development, ovarian follicular growth, oocyte maturation), transcription, synthesis of proteins, and DNA replication ( Figure 1). ^9^ Formation of Zn finger (Znf) motifs helps many transcription factors to bind DNA. Zinc is involved in bone formation, while collagen in the bone prevents bone resorption by stimulating alkaline phosphatase (ALP) activity in the osteoblasts. ^10^ Zinc influences many antioxidant enzymes, including Cu/Zn superoxide dismutase (SOD1), which prevents DNA damage and aids cell sensitivity towards insulin. Zinc stimulates growth promotion by stimulating growth hormone secretion, insulin like growth factor (IGF-1), insulin-like growth factor binding protein (IGFBP), and IGF-3 secretion. Insulin-like growth factor1(IGF1) promotes differentiation of the placenta, placental growth, and functional development in pregnancy during its early stages. ^10^^–^^16^

### Copper

**Figure 2.**
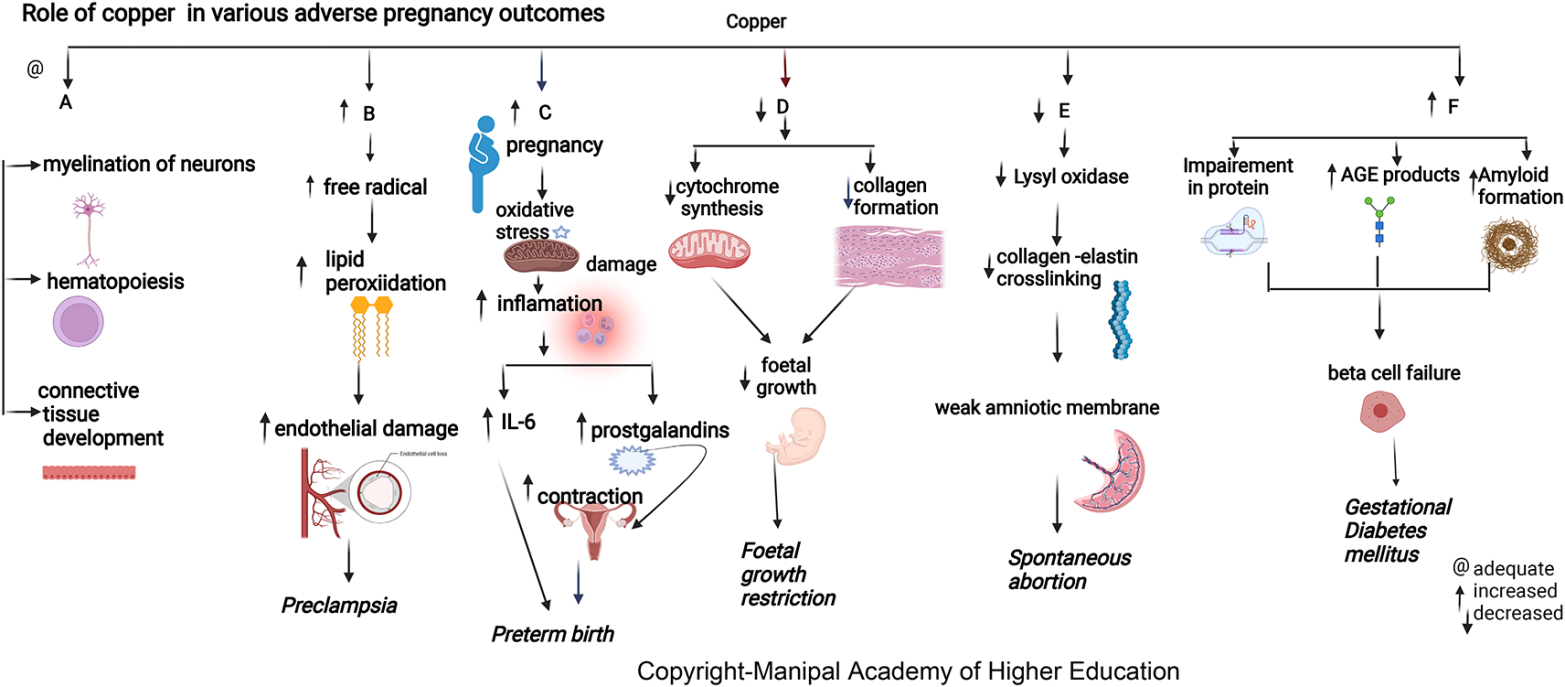
A-Copper is biologically important for myelination of neurons, hematopoiesis and connective tissue development. B-Increased copper contribute to disease progression by producing free radicals that lead to lipidperoxidation and damaging endothelial cell function leading preeclampsia. C-Elevated plasma copper in early pregnancy may indicate inflammation that may predispose to preterm delivery. D-Copper deficiency can hamper cytochrome and bonecollagen synthesis, directly affecting fetal growth leading to Fetal growth restriction. E-Copper is involved in placentation, Lysyloxidase's unavailability due to copper deficiency can reduce collagen and elastin crosslinking, leading to the weak amniotic membrane and spontaneous abortion. F-Copper binds nucleicacids and proteins, it plays a role in protein crosslinking, and generation of advanced glycation endproducts leading to their functional impairment.

Copper is biologically essential for oxidation, energy metabolism, myelination of neurons, hematopoiesis, iron transport, and defense against free radicals. ^14^ The maternal copper level increases during pregnancy due to an increase in ceruloplasmin induced by increased estrogen. Copper in cytochromes helps in electron transport and energy generation. Copper (I) ions can produce reactive oxygen species (ROS) ( Figure 2), catalyze the Haber–Weiss reaction, and induce insulin resistance by ROS leading to altered glucose homeostasis. ^9^^,^^10^^,^^15^^,^^16^ Lysyl oxidase (LOX), a copper-containing enzyme, is involved in oxidizing lysine and hydroxylysine residues present in precursors of elastin and collagen, also enabling crosslinking in the amniotic membrane. This crosslinking maintains mature elastin’s elasticity and stabilizes collagen fibrils. ^18^

### Magnesium

**Figure 3.**
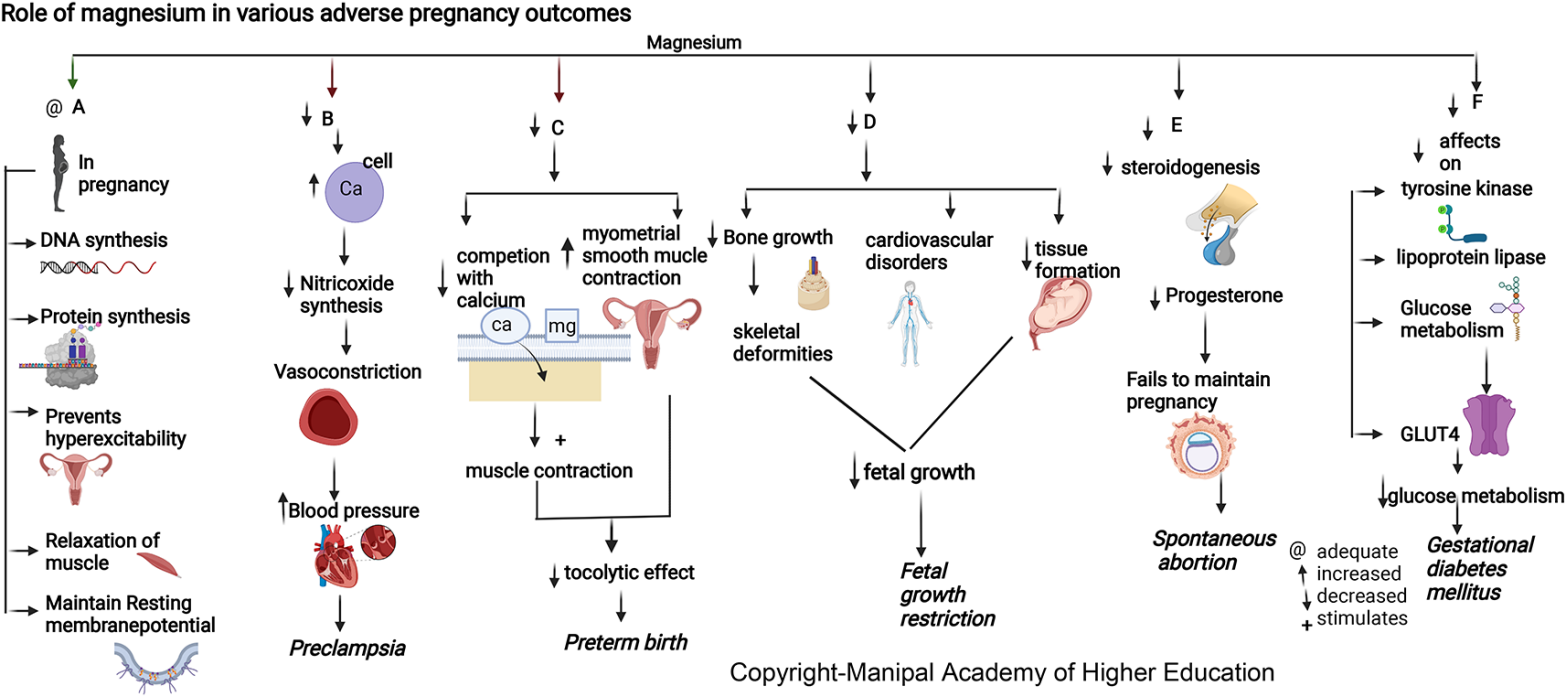
A-In pregnancy magnesium is involved in synthesis of DNA and proteins, prevents uterine hyperexcitability, relaxation of muscles and involved in resting membrane potential. B-Hypomagnesemia leads to increased intracellular calcium and a reduction in nitric oxide synthesis causing vasoconstriction and elevated blood pressure. C-Decreased magnesium will reduce the competition with calcium stimulating muscle contraction and increase myometrial smooth muscle contraction in uterus causing reduced tocolytic effect leading preterm birth. D-Decreased magnesium is associated with decreased bone growth, skeletal deformities, cardiovascular disorders and decreased tissue formation in foetus leading to foetal growth restriction. E-Magnesium deficiency decreases steroidogenesis (particularly progesterone synthesis) required for maintenance of pregnancy, leading to spontaneous abortion. F-Magnesium deficiency has decreased effects on tyrosinekinase, glucose transporter 4, lipoprotein lipase, decreased glucose metabolism leading Gestational diabetes mellitus.

Magnesium is involved in >600 enzymatic reactions, such as energy metabolism, fatty acid and protein synthesis, neuromuscular excitability, and uterine hyperexcitability prevention in pregnancy, relaxation of muscles, vasodilatation and decreased vascular resistance, and transmission of nerve impulses ( Figure 3). Magnesium stimulates the activation of vitamin D, thus involved in the homeostasis of calcium and phosphate, and is essential for calcium-triggered release of parathyroid hormone (PTH). Magnesium acts as an allosteric activator for adenylate cyclase, Na/K-ATPase, and phospholipase C. Magnesium downregulates IL-1 alpha, IL-4, IL-6, IL-1 beta, Il-10, IL-12, TNF-alpha and several chemokines involved in hypertension, numerous immune-inflammatory pathways, and in adverse pregnancies. Magnesium protects against pregnancy inflammation by nitric oxide synthase enzyme inhibition. It decreases proinflammatory cytokine production of activated B cell receptors by blocking the nuclear factor kappa-light-chain-enhancer. ^19^^–^^21^

### Selenium

Selenium plays a role in antioxidative protection, protein synthesis, enzyme function, formation of thyroid hormones, and immunomodulatory and anti-proliferative mechanisms, all of which impact pregnancy. For adequate functioning of enzymes, selenium concentrations have to be about 80–95 μg/L plasma, corresponding to ~100–120 μg/L in the whole blood. It is a vital component of the selenoproteins and glutathione peroxidase and is involved in periconceptional events such as oocyte development, fertilization, follicle growth, maturation, and implantation. Selenium, through selenoproteins like DIO2, GPX1, and selenoprotein K, plays a role in proper functioning of trophoblasts during pregnancy and an index of oxidative stress, activating signaling pathways such as phosphoinositide 3-kinase activation (PI3K/AKT) and extracellular-signal-regulated kinase (ERK). Morphological changes in the fetal cerebellum correlate with selenium levels during pregnancy. Se involves neonatal cerebellum measures, length and width, and children’s motor and cognitive scores. ^15^^,^^21^^,^^22^ Selenium can alleviate the inflammatory response, which attenuates insulin resistance by inhibiting the NF-κβ signaling pathway and TLR through the expression of PPAR-γ and glucose homeostasis throughout pregnancy by enhancing glucose transporters’ activity (GLUT) and neutralizing reactive oxygen and nitrogen species.

### Iron

**Figure 4.**
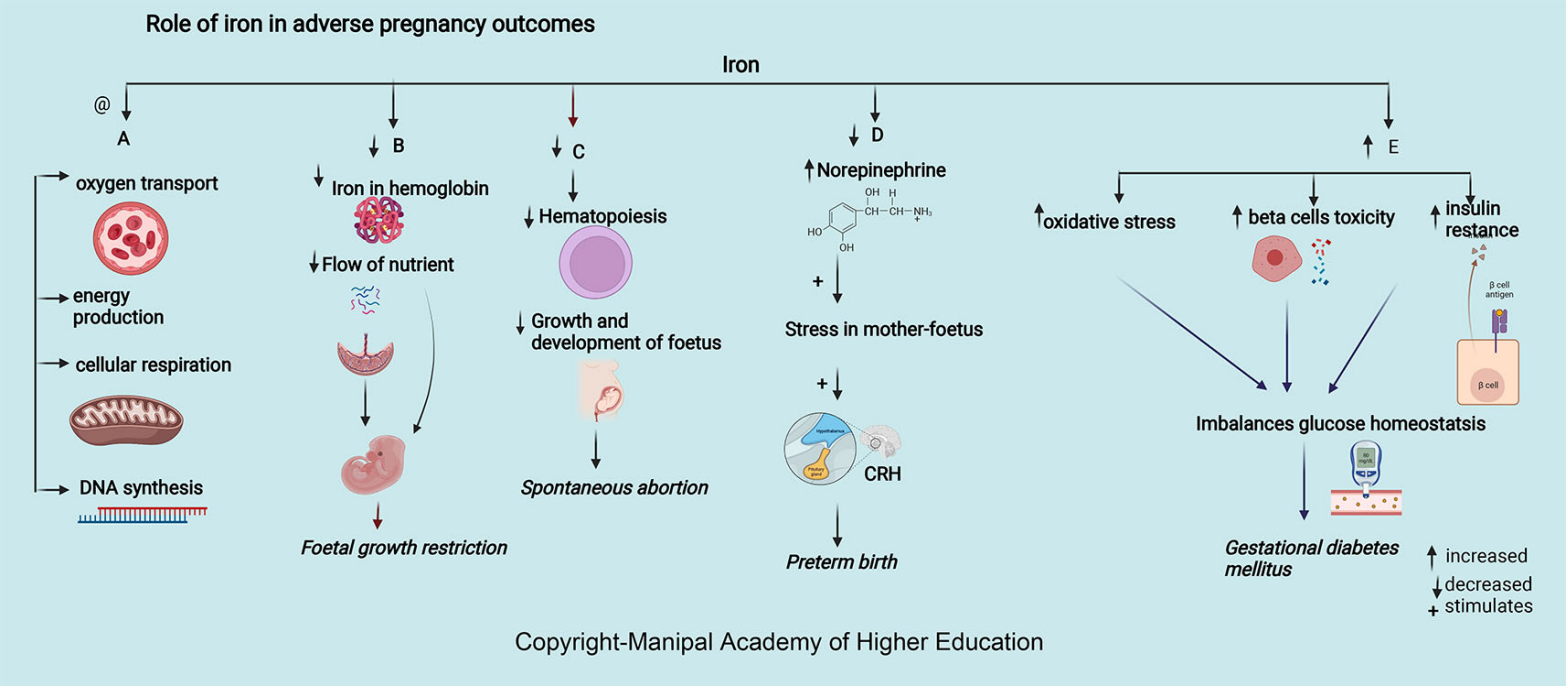
A-Adequate iron is involved in oxygen transport, energy production, cellular respiration, and DNA synthesis. B -Decreased Iron in haemoglobin delivers low oxygen through the maternal-placental-foetal unit leading foetal growth restriction. C-Decreased Iron leads decreased haematopoiesis, growth, and foetus development leading spontaneous abortion. D-Decreased iron increases the chances of preterm by increasing serum norepinephrine concentrations, which may induce maternal and foetal stress, stimulate the synthesis of corticotropin-releasing hormone (CRH). E-Excessive iron levels increase oxidative stress, beta cell toxicity and insulin resistance disrupting glucose homeostasis causing gestational diabetes mellitus.

Iron is integral to the transport of oxygen, energy production, cellular respiration, and DNA synthesis. Iron in ferritin prevents cellular damage and is critical to normal β cell function and glucose homeostasis ( Figure 4). Iron in hemoglobin delivers oxygen through the placental unit to maintain the oxygen requirement of a fetus, of which 60% is utilized mainly for developing the nervous system to a complex sulcated and gyrated brain structure of the fetus. The homeostatic response of changes in iron protects the placenta; for example, in iron deficiency, i.e., iron import increases through transferrin receptor 1 (TFR1), and export through ferroportin (FPN) decreases. ^23^ Excessive iron is toxic and free iron can lead to oxidative stress and inflammation. In severe cases, it can lead to ferroptosis and cell death by lipid peroxidation of cell membrane. High levels of proinflammatory cytokines in maternal serum in pregnancy maintain adequate placentation, leading to inflammation and a mild systemic inflammatory response. Interleukin 6 (IL-6), the primary hepcidin regulator, disturbs iron homeostasis and leads to the expression of vascular cell adhesion molecule (VCAM1) and intercellular adhesion molecules (ICAM-1), maintaining endothelium adhesive phenotype. ^17^^,^^23^^–^^29^

## Spontaneous abortion

World Health Organization (WHO) defines SA as the termination or removal of a fetus of weight ≤ 500 grams before the 20 ^th^ week of gestation. ^21^^,^^27^ The incidence of spontaneous miscarriage is 10–30% worldwide of clinically diagnosed pregnancies, of which 80% are within 12 weeks of pregnancy. Studies reported that the altered concentration of serum micronutrients (Zn, Cu, Se, Cd, Fe, and Mg) might lead to spontaneous abortions. ^28^

### Zinc

Zinc helps in embryo development and building up of maternal immunity. Mothers who present with spontaneous abortion have significantly lower mean concentrations of serum zinc. ^14^^,^^16^^,^^28^^–^^30^ Low serum zinc levels can lead to decreased cell generation, reduced protein synthesis, and increased cellular oxidative stress ( Figure 1). Insufficient supply of antioxidants in maternal and placental circulation can predispose to complications in pregnancy. ^31^ Zinc deficiency decreases antibody production, affecting maternal and fetal immunity, causing spontaneous abortion. ^32^ Many studies have shown that zinc supplementation leads to altered thymus gland secretions leading to a shift in T-helper cells. All these can predispose to recurrent spontaneous abortion in women. ^16^

### Copper

Copper has roles in connective tissue development, hematopoiesis, energy metabolism, neurotransmitter synthesis, and myelination of neurons. ^30^ Serum copper levels are 30% to 35% lower in women with miscarriages. ^29^ Low maternal serum copper around 15 weeks (about three and a half months) of gestation has a protective effect against SA, showing that copper is more involved in placentation than conception. In addition to other factors, optimum serum copper concentration at 15 weeks of gestation decreases spontaneous abortion. ^17^ Low maternal serum copper levels after 28 weeks (about six and a half months) of pregnancy were associated with higher rates of premature rupture of membranes (PROM). ^33^ Lysyl oxidase’s unavailability due to copper deficiency can reduce collagen and elastin crosslinking, leading to a weak amniotic membrane in a spontaneous abortion case. But copper supplementations in pregnancy did not show the reversal of this effect. ^31^ During pregnancy, changes in ceruloplasmin (copper-containing protein) levels can significantly change the ability of neutrophils to produce reactive oxygen species (ROS), which increases the chances of SA. As the metabolism of copper and iron is interlinked, deficiency of iron results in an elevation in liver copper. The above mechanism is linked with an elevated maternal serum copper and that of serum ceruloplasmin. ^33^

### Selenium

Selenium, due to its antioxidant activity and involvement in the immune system, helps prevent heart diseases and cancer. ^34^^,^^35^ Selenium deficiency causes decreased immune system activity, affecting both innate and adaptative immune responses. Selenium-associated enzymes such as thioredoxin reductases and glutathione peroxidases prevent reactive oxygen species-induced cell damage. Adequate selenium is responsible for improving the activation of natural killer (NK) cells and T cells. The lower levels of antioxidants due to reduced Se disturb the balance of thromboxane-prostacyclin, leading to SA. However, this has not been explored in-depth and can be studied further in the future. In pregnant women, an increased Se demand due to increased erythropoiesis in the growing fetus must be met to prevent abortion. ^19^^,^^30^

### Magnesium

Magnesium (Mg) is involved in maintaining body temperature, synthesis of nucleic acid and protein ( Figure 3), and maintenance of resting membrane potential in the nerve and muscle cells. ^36^ During pregnancy, the maternal plasma magnesium increases with gestational age. Low antenatal Mg with an increased Ca/Mg ratio may lead to spontaneous abortions. ^28^ It plays a significant role in membrane transport such as transmembrane electrolyte flux, transport of potassium and calcium ions across the plasma membrane by active transport mechanism involved in maintaining the normal functioning of vital organs such as fetal cardiac activity in the first trimester, maintenance of cell adhesion and cell migration, and neurochemical transmission involved in feto-maternal circulation. Hence, magnesium deficiency may hamper further growth and functioning of fetal organs, leading to SA. Magnesium functions in steroidogenesis (particularly progesterone synthesis) but is also involved in the maintenance of pregnancy is not clearly defined by the researchers. Studies can explore this area of the mechanism involved in the etiology of the same. ^14^^,^^30^

### Cadmium

Ingested cadmium can selectively accumulate in the brain, red blood cells (RBC), kidneys, liver, and bones leading to its toxicity. This metal can displace other metals from metalloenzymes. It can seriously impact various organ morphologies and can modulate the functions of iron, calcium, copper, zinc, manganese, magnesium, etc. As living organisms poorly eliminate cadmium, it has a biological half-life of 16-30 years. ^36^ At 12-week pregnancy, blood cadmium >0.4 μg/L can increase the risk of embryotoxicity and spontaneous abortions.

### Iron

Iron is essential in pregnancy to meet the increased demands for hematopoiesis, fetus development, and growth ( Figure 4). 42% of children below five years, 50% of pregnant women-, and 33% women of reproductive age are iron deficient worldwide. ^37^^,^^38^ Among women with miscarriages, 13% higher serum iron levels are seen in 14 weeks of pregnancy. In support of the above findings, serum hepcidin and ferritin concentrations were higher in the first trimester due to possible accumulation of iron in maternal serum because the fetus is not receiving maternal iron through the placenta leading to SA. ^39^ Iron supplements during an antenatal period can significantly minimize the incidence of SA. ^40^

## Preterm birth

Preterm is the birth of a live baby <37 weeks of gestation. ^30^ Reduced concentration of collagen, collagen crosslinking, increased oxidative damage biomarkers, and inflammatory signals transmitted in the placental membrane resulting from cellular apoptosis stimulate parturition in pregnancy leading to preterm delivery. ^34^ Copper, zinc, manganese, and selenium counterbalance the oxidative stress-induced premature aging of the placenta and inflammatory responses. ^30^

### Zinc

Approximately 18% of zinc deficiency during pregnancy causes adverse effects, leading to premature birth, increased infections, and nanism in children. ^41^ Serum zinc concentration tends to decline in early pregnancy and reduces to approximately 35% less than the levels in non-pregnant women. Zinc is present as the highest content in the brain involved in migration and differentiation of the nervous system, i.e., neurogenesis ( Figure 1). More than 50% of cerebral white matter injury (WMI) in premature birth is attributed to zinc deficiency. ^42^ Zinc and other micronutrients maintain the birth length (BL) and fetal birth weight (BW) and are involved in the differentiation of oligodendrocytes, which contribute to the onset of myelination. The head circumference of the fetus is the crucial factor in deciding the week of delivery. ^32^ The increased risk of amniotic infection by inhibiting maternal and fetal immunological competence due to lower maternal serum zinc concentration may lead to preterm birth. Thus, maintaining an adequate serum zinc concentration during pregnancy may help keep optimal fetal growth and prevent preterm birth. ^35^^,^^41^^,^^44^

### Copper

Both copper deficiency and excess can lead to adverse pregnancy outcomes. ^43^ Elevated plasma copper in early pregnancy may indicate inflammation predisposing to preterm delivery. ( Figure 2) During pregnancy, estrogen mediates an increase in copper-carrying proteins in the blood, which may lead to a rise in circulating levels of copper. A low copper/zinc ratio reduces the risk of pregnancy complications. Studies show that lower serum copper has a protective effect early as the mature fetal liver stores the trace elements. ^43^ Hence, the maternal serum delivers higher copper in preterm delivery as its fetal transfer remains incomplete due to reduced pregnancy duration compared to term pregnancy.

### Iron

Iron deprivation in the first and second trimesters of pregnancy can lead to premature birth and affect the baby’s health. ^44^ During pregnancy, decreased iron and anemia increase serum norepinephrine concentrations, induce stress in mother and fetus, leading to corticotropin-releasing hormone (CRH) synthesis, and increase the chances of preterm. Increased maternal infection due to increased oxidative damage to RBC and the placental unit can result in preterm delivery. ^47^^,^^48^ The requirement for iron ranges from 1–1.5 mg/day and increases to 5.0 mg/day in the last two trimesters. Due to the expansion of maternal blood volume, third-trimester pregnancy needs more iron. The risk of preterm term birth has shown an inverse correlation with the duration of iron supplementation. As iron inadequacy leads to functional impairment and excess leads to cytotoxicity, the dose of iron supplementation remains debatable; hence further studies need to focus on the amount and duration of supplementation. ^45^^,^^46^^,^^49^

### Magnesium

Many studies reported a reduced incidence of preterm delivery with magnesium given to the mother <12 hours before delivery. ^47^^,^^48^ PROM has been linked with lower serum magnesium in mothers and higher in the placenta, implying distinct pathophysiological roles for magnesium compared to preterm birth ( Figure 3). Magnesium has the ability to compete with intracellular calcium in maintaining the membrane potential in preterm labor. ^52^ Magnesium protects against premature birth by inhibiting myometrial activation and cervical maturation. ^52^^–^^54^

## Fetal growth restriction

Twenty million infants globally are born as low birth per year weight babies (<2500g). Among these, approximately 95% are from developing nations. In India, fetal growth restriction is about 18 to 20%, primarily due to maternal micronutrient deficiency. ^51^^–^^53^

### Zinc

Based on studies conducted in both animal models and humans in pregnancy, decreased maternal zinc is associated with fetal growth restrictions. Mothers are 2.6 times more likely to have FGR with low zinc levels. ^52^ The zinc levels are significantly lower in mothers with BMI less than 18.5 who had small gestation (SGA) neonates. ^20^^,^^50^^,^^52^ Maternal zinc deficiency in early pregnancy increases the risk for FGR ( Figure 1) as many enzymes and growth hormones, which play a role in fetal growth, require zinc. In FGR, osteoblast formation *via* IGF-I in the fetus gets affected due to zinc deficiency, as explained above in the metabolic functions. ^56^ Studies have shown that with maternal zinc supplementation, a substantial number of babies are born with appropriate birth weight, indicating that monitoring serum zinc in pregnancy is essential to ensure the proper development of the fetus and prevent FGR. ^50^

### Copper

Copper protects cells from toxic superoxide anions to ensure average fetal growth and immune function. It also maintains the hematopoietic, central nervous, bone, and connective tissues. Maternal serum copper has an inverse relation with head circumference (HC), body weight, and the fetus’s height ( Figure 2). ^54^ Studies indicate that pregnant women with a deficiency of copper are prone to fetal growth restriction (FGR) as it affects collagen and elastin metabolism involved in maintaining average fetal growth (mechanism as discussed above in the metabolic section). Copper deficiency can hamper cytochrome and bone collagen synthesis, directly affecting fetal development and leading to FGR. ^52^^,^^55^^,^^59^^–^^61^

### Iron

Pregnancy with iron deficiency reduces fetal growth ( Figure 4) and affects neuronal growth, especially in the myelinization of neurons, neural signals transmission, frontal cortex development, and basal ganglia. ^26^^,^^56^ Decreased iron leads to decreased hemoglobin, restricting oxygen circulation and forming an environment of chronic hypoxia, leading to fetal growth restriction. Iron deficiency stimulates the synthesis of corticotropin-releasing hormone *via* increased norepinephrine and, in turn, hinders the growth of the fetus. Studies have shown that prenatal iron supplementation had a beneficial effect on baby weight in women who had taken Fe supplements during the last two trimesters of pregnancy. ^62^ Although precise mechanisms are not clear on iron-folic acid and multiple-micronutrient supplements preventing FGR, few studies states that it maintains the average plasma volume and the flow of nutrients across the placental tissue to the fetus. ^62^^–^^64^

### Magnesium

Mother and fetus tissue formation in pregnancy requires a high magnesium intake. ^53^^,^^65^^,^^66^ Magnesium is involved in vitamin D activation and in maintaining calcium and phosphate physiology for bone growth. Dysregulation in either of these nutrients is associated with skeletal deformities and cardiovascular disorders, leading to fetal growth restriction. Studies have shown that magnesium supplementation to pregnant mothers can suppress the chance of intrauterine growth restriction (IUGR), optimum birth weight of baby, head circumference, and body height that can be attributed to alterations in cytokine/chemokine in the amniotic fluid and placenta. ^66^

## Gestational Diabetes Mellitus (GDM)

Gestational diabetes mellitus is glucose intolerance at any level at the beginning of pregnancy. The high plasma fasting glucose levels may increase the chances of fetal deaths in the last weeks of gestation. Obesity and sedentary lifestyles are the main factors, and dietary intake is essential in the progression of GDM.

### Zinc

Among GDM cases, there are low serum Zn levels in the second and third trimesters. Giving zinc and calcium- magnesium and vitamin D supplements for six weeks in the second trimester to subjects with GDM improves serum insulin, fasting plasma glucose levels, and the homeostatic model of measuring insulin resistance. ^58^^,^^67^ Zinc intake improved fasting plasma glucose (FPG) and lipid profiles. Zinc has a role in enzymes involved in the metabolism of lipid-like action on insulin. GDM occurs *via* oxidative stress and trace elements’ effects on immunomodulation and insulin activity changes. The immunomodulation mechanisms in GDM are related to oxidative stress and the impact of trace elements on the regulation of the immune system. Oxidative stress induces apoptotic deletion in the regulatory T cells, which are CD8+CD25+activated in the GDM. Besides, the Zn-insulin complex is a hexamer containing two zinc ions and forms other complexes with insulin. Therefore, zinc supplements may benefit glucose homeostasis. ^58^^,^^68^

### Copper

Copper has shown positive correlations with HbA _1C_ in types 1, 2 diabetes and GDM. ^70^ Serum copper levels are directly involved in developing GDM independent of other established risk factors. Copper directly binds proteins affecting protein crosslinking, leading to the generation of advanced glycation end products, resulting in its functional impairment. Copper can promote progressive islet b-cell failure by causing the clustering of human amylin into an amyloid fibril. The hydrogen peroxide formed in the above process could also stimulate progressive b-cells degeneration. ^67^^,^^68^^,^^72^ In contrast to the above finding, in underweight Chinese women, higher maternal serum copper has decreased the GDM risk before 24 weeks as the body composition, and body mass index (BMI) are associated with the individual trace-element status. ^68^^,^^69^

### Magnesium

Maternal serum magnesium is low in the second and third trimesters with gestational diabetes mellitus. ^71^ Insulin resistance might increase the placental size leading to low placental efficiency and affect fetal growth in GDM patients. ^72^ Magnesium, by its effects on tyrosine kinase, glucose transporter 4, and lipoprotein lipase, has a significant role in glucose metabolism ( Figure 3). Supplementing magnesium and vitamin E in GDM patients resulted in a significant improvement in glycaemic control and lipid profile. ^71^^,^^73^

### Iron

Maternal serum ferritin > 67.8 μg/L and iron levels 52.9 mmol/L at 12 weeks of gestation are strong predictors for developing GDM as excessive iron levels increase oxidative stress, beta cell toxicity, and insulin resistance ( Figure 4), disrupting glucose homeostasis ^67^^,^^74^ Higher HbA1C levels noticed among individuals of iron deficiency have been detected. ^75^ Iron supplementation has not been recommended for non-anaemic pregnant women in the first and second trimesters as it could elevate post-prandial blood glucose and cause GDM. ^19^^,^^74^^,^^76^^,^^77^

## Preeclampsia

Preeclampsia is hypertension in pregnancy with proteinuria of at least 0.3g/day in 2 to 8% of pregnant women and is the reason for about 63,000 deaths every year worldwide, especially in Nigeria and India ^78^ Worldwide, PE management is still the leading cause for maternal and fetal mortality. ^6^^,^^13^ Several micronutrients such as zinc, copper, magnesium, and calcium abnormalities play a role in preeclampsia. ^42^^,^^55^^,^^75^^,^^79^

### Zinc

Many studies worldwide associated low maternal serum zinc in PE independent of geographical location. Albuminuria in PE predisposes to zinc deficiency leading to decreased delivery to cells. Reduced serum Zn concentrations in preeclampsia reflect reduced Zn binding protein and estrogen concentration. ^55^^,^^79^ Low zinc levels in pregnancy can increase blood pressure by reducing the amount of calcium in the muscles and decreasing the excretion of sodium in the urine due to its effect on sodium chloride cotransporter in the kidney leading to PE. ^82^^,^^83^ Zinc deficiency reduces NOS activity contributing to hypertension development by dysfunction in the endothelium, and reduces vasodilation, an endothelium-mediated activity. Zn, a signaling molecule, controls receptor and growth factor-mediated pathways in the cell’s immunological mechanisms. ^80^ In addition, Zinc deficiency contributes to oxidative damage by causing a negative effect on the copper-zinc superoxide dismutase enzyme system. ^81^ Lipid peroxides and antioxidants play a vital role in preeclampsia. Reduced zinc and increased Cu/Zn ratios lead to PE by inactivating antioxidant enzymes causing inadequate trophoblast invasion, suboptimal perfusion in the placenta, and ischemia. ^83^^,^^84^

### Copper

In pregnant women, the lower prevalence of PE observed with high copper signifies its importance in the pathogenesis of preeclampsia. ^6^^,^^7^^,^^80^ Copper levels increase under estrogenic activity, the main determinant of ceruloplasmin expression. Copper has a predictive cut-off value of 224μg/dL, indicating a good biomarker for PE diagnosis. Copper contributes to disease progression by lipid peroxidation, free radicals, and damaging endothelial cell function. ^84^^,^^85^ Copper and Ceruloplasmin (cp) have a role in deciding the termination of preeclamptic pregnancies under 34 weeks (about eight months), as the copper and cp levels have shown a linear increase according to the severity of preeclampsia. ^83^ Copper has the highest pro-oxidant capacity and serves as a cofactor to superoxide dismutase. ( Figure 2). ^13^^,^^27^ Since preeclampsia is a proteinopathy disorder with impaired autophagy, protein aggregates (Aβ, TTR, P-tau231, and α-syn) are generated by impaired autophagy in the placenta or kidneys. ^82^ In PE, the lipid peroxidation product, malondialdehyde, and altered amyloid-b production generate protein aggregates, toxic amyloid-b42 species, and their specific components α-syn, TTR, P-tau231, and Aβ. ^82^

### Magnesium

During pregnancy, a suitable magnesium level of 360–400 mg/day and reduced serum magnesium levels can trigger preeclampsia. ^84^ Magnesium influences the vascular tone and contractility and plays a vital role in blood pressure regulation ( Figure 3). During pregnancy, an ionized fraction of magnesium of less than 24.67% can predispose to preeclampsia. This forms the basis for using magnesium sulfate therapy for preventing and treating seizures associated with preeclampsia/eclampsia. ^69^^,^^75^^,^^76^ Hypomagnesemia leads to intracellular calcium influx and a reduction in nitric oxide synthesis, causing vasoconstriction and elevated blood pressure. ^6^^,^^53^^,^^69^^,^^79^ Hence Magnesium gluconate (3 g/day) has been used in managing pregnancy hypertension in high-risk pregnant women. ^86^

### Iron

Increased iron, systemic inflammation markers, and superoxide status are associated with preeclampsia. In PE, systemic inflammation and concentrations of superoxide are higher and result in endothelial function deterioration. A 2.19-fold risk of pregnancy-induced hypertension was seen in the lowest serum iron level (≤801.20 μg/L) at 10-14 weeks (about three months) of pregnancy as it affects trophoblast development. Reduced iron causes abnormal invasion of trophoblasts in the walls of the spiral arteries (between 6–18 weeks, about four months of pregnancy), resulting in reduced high-resistance circulation in the uteroplacental unit re-modeling, hypoxia, increased apoptosis, activation of the inflammatory and immune response. All these can lead to vascular endothelial damage and disturbance in endothelial homeostasis resulting in vasoconstriction and increased blood pressure. ^26^ In the third trimester of pregnancy, there was a positive correlation between ferritin, iron, and superoxide concentrations with vascular stiffness. The World Health Organization recommends universal iron supplementation of 30–60 mg/day throughout pregnancy. There is an increased risk of hypertension after 20 weeks of pregnancy with iron supplementation before 16-week as it causes inhibition of lymphocyte proliferation, development of oxidative stress, and elevated blood viscosity. ( Figure 4) ^87^^,^^88^ Hence supplementation of iron along with vitamin E and antioxidant-rich foods is advisable only for pregnant women with proven iron deficiency to prevent iron overload. ^25^^,^^89^^,^^90^

### Calcium

Hypocalcemia in pregnancy has seven times more risk of developing preeclampsia. A dietary deficiency in calcium may cause decreased calcium in all membrane storage sites, resulting in reduced stability in the cell membrane of vascular smooth muscle. Calcium works with magnesium in maintaining the equilibrium in the ions within the vascular membrane, thus affecting blood pressure. ^77^^,^^84^^,^^85^^,^^91^ Hypocalcemia or hypovitaminosis D triggers the secretion of parathyroid hormone and renin, increasing intracellular calcium concentration and leading to vasoconstriction. ^74^^,^^81^ Hence, the World Health Organization has recommended supplementation of calcium to prevent preeclampsia in pregnant women with lower intake.

### Selenium

Selenium is an essential component of selenoenzymes and placental protection against oxidative stress. Selenium status is most important at 8–10 weeks (about two and a half months) gestation when there is an initiation of intervillous blood flow, posing an oxidant challenge. ^92^ The high prevalence (56.9%) of serum selenium deficiency in the preeclamptic patient suggests that selenium deficiency is a risk factor for the development of preeclampsia and reported a serum selenium level of less than 50μg/l (<0.633μmol/l) in 62% of the preeclamptic patients. ^57^ A deficiency of selenium could lead to trophoblast cell function disorders and arouse endothelial cell activation, along with free radicals, which expedite maternal vascular malfunction, which predisposes to PE. Low levels of selenium lead to decreased glutathione peroxidases (GPx), thioredoxin reductases (Th-Redx), and selenoproteins P, antioxidative and anti-inflammatory selenoproteins needed to protect fetal, placental, and maternal tissue against oxidative stress and damage. Activating oxidative stress markers, inflammatory cytokines, fibronectin, endothelin 1, nitric oxide deficiency, prostacyclin, and vasodilators increase blood pressure. ^93^ Selenium supplementation can influence preeclampsia risk in populations with lower selenium status. ^93^

## Conclusion

Micronutrients play multiple roles during pregnancy and fetoplacental growth like stimulating growth hormone secretion IGF-1, IGFBP, and IGF-3 secretion, leading to growth promotion, Lysyl oxidase (LOX), oxidizes lysine and hydroxylysine residues present in the precursors of elastin and collagen, enabling crosslinking in the amniotic membrane, downregulation of IL-1 alpha, IL-1 beta, IL-6, IL-4, IL-12, Il-10, TNF-alpha and several chemokines involved in hypertension, numerous immune-inflammatory pathways, fetal cerebellum morphological changes, attenuate insulin resistance and improve the activity of glucose transporters, structural development of the nervous system of the fetus, dysfunction in placenta and injury to trophoblast through hypoxia-reperfusion. Over supplementation had led to complications such as spontaneous abortion, and gestational diabetes mellitus. So, as there is a lack of standardization concerning micronutrient supplementation during pregnancy, there is a need for systematic study related to the role of micronutrients during each trimester of pregnancy to optimize its supplementation and to prevent hazards associated with its abuse

## Data Availability

No data are associated with this article.
