# Supplementary material for: Micronutrients in Adverse Pregnancy Outcomes
Source: F1000Res. 2024 Jun 21;11:1369. Originally published 2022 Nov 23. [Version 4] doi: 10.12688/f1000research.124960.4 (PMC11131564; doi:10.12688/f1000research.124960.4)
Supplement: Peer review history

# Reviewer report: Reviewer response for version 4

**DOI:** 10.5256/f1000research.167997.r293774

**Published:** 2024-06-24

**Authors:** Giovanni Tossetta

**Affiliations:** 1 Università Politecnica delle Marche, Ancona, Italy

The manuscript has been significantly improved and can be accepted in the present form

Is the review written in accessible language?

Yes

Are all factual statements correct and adequately supported by citations?

Yes

Are the conclusions drawn appropriate in the context of the current research literature?

Yes

Is the topic of the review discussed comprehensively in the context of the current literature?

Yes

Reviewer Expertise:

Pregnancy complications

I confirm that I have read this submission and believe that I have an appropriate level of expertise to confirm that it is of an acceptable scientific standard.

# Reviewer report: Reviewer response for version 3

**DOI:** 10.5256/f1000research.162687.r281711

**Published:** 2024-05-25

**Authors:** Giovanni Tossetta

**Affiliations:** 1 Università Politecnica delle Marche, Ancona, Italy

the manuscript is interesting and generally well written and illustrated. Figures are clear and easily understandable. Moreover, manuscript has been significantly improved after revision.

 However, two points could be improved. 

**Introduction**: this section could be improved since it is too superficial for a review article. In fact, it could be highlighted that oxidative stress and inflammation are two common characteristics of the pregnancy complications treated in this manuscript (see for example PMID: 33477354, PMID: 37296665). 

**A table summarizing the main results of the studies treated would be helpful**

Is the review written in accessible language?

Yes

Are all factual statements correct and adequately supported by citations?

Yes

Are the conclusions drawn appropriate in the context of the current research literature?

Yes

Is the topic of the review discussed comprehensively in the context of the current literature?

Yes

Reviewer Expertise:

Pregnancy complications

I confirm that I have read this submission and believe that I have an appropriate level of expertise to confirm that it is of an acceptable scientific standard.

## References

[1] : Modulation of NRF2/KEAP1 Signaling in Preeclampsia. *Cells* .2023;12(11) : 10.3390/cells12111545 10.3390/cells12111545 37296665 PMC10252212

[2] : The Multifaced Actions of Curcumin in Pregnancy Outcome. *Antioxidants (Basel)* .2021;10(1) : 10.3390/antiox10010126 10.3390/antiox10010126 33477354 PMC7830020

# Response

**Published:** 2024-05-27

**Authors:** Krishnananda Prabhu

**Affiliations:** Kasturba Medical College, Manipal, India

Dear Sir/Madam,

 Thank you very much for your valuable inputs in enriching this article. We shall incorporate the suggestions soon and resend

# Reviewer report: Reviewer response for version 1

**DOI:** 10.5256/f1000research.137211.r199841

**Published:** 2024-01-19

**Authors:** Jitendra Ahuja

**Affiliations:** 1 Rajasthan University of Health Sciences, Jaipur, Rajasthan, India

Relevance and Description:

 The article addresses a pertinent and current issue as adverse pregnancy outcomes remain a significant concern in the realm of maternal and fetal health. Micronutrient supplementation during pregnancy is a common practice, but the absence of standardized guidelines has raised concerns about potential overuse and its consequent adverse effects. Grasping the role of micronutrients during pregnancy and their connection to complications is pivotal for enhancing the well-being of both mother and fetus.

 Major Points:

**Clear Problem Statement:** The article adeptly elucidates the issue of adverse pregnancy outcomes and their correlation with maternal nutritional adjustments. It underscores the gravity of this problem, given that approximately 10 to 20% of pregnancies are affected by complications.

**Significance of Micronutrients**: The article offers a comprehensive overview of the multifaceted roles played by micronutrients during pregnancy. It delves into how these micronutrients influence a variety of physiological processes, including hormone secretion, collagen and elastin crosslinking, immune responses, and neurodevelopment.

**Overuse of Micronutrient Supplementation**: The article correctly identifies the absence of well-defined guidelines as a major concern. It highlights that the excessive or inappropriate utilization of multiple micronutrient supplements during pregnancy has resulted in adverse outcomes, such as spontaneous abortion and gestational diabetes mellitus.

**Need for Systematic Studies:** The article rightly emphasizes the necessity for systematic research to optimize micronutrient supplementation during each trimester of pregnancy. Such an approach could potentially avert complications associated with both deficiency and excessive supplementation.

**Minor Points:**

 The article could be enhanced by a more extensive discussion of specific micronutrients and their distinct impacts on pregnancy outcomes. This could provide a more nuanced comprehension of the subject matter.

 While the article mentions the sources it utilized, a dedicated section detailing the methodology, encompassing the search strategy and inclusion/exclusion criteria for the considered studies, would enhance clarity.

**Overall Opinion:**

 This article constitutes a valuable addition to the field of maternal and fetal health by addressing the critical concern of micronutrient supplementation during pregnancy and its potential consequences on adverse outcomes. The problem is elucidated effectively, and the importance of addressing it is manifest. The article furnishes a comprehensive overview of the roles of micronutrients and appropriately underscores the necessity for systematic studies and standardized guidelines.

 To further enrich the article, a more in-depth exploration of individual micronutrients and a more transparent presentation of the research methodology employed for the literature search would be beneficial. In summation, this work is informative and holds promise in guiding future research and clinical practices in maternal nutrition.

Is the review written in accessible language?

Yes

Are all factual statements correct and adequately supported by citations?

Yes

Are the conclusions drawn appropriate in the context of the current research literature?

Yes

Is the topic of the review discussed comprehensively in the context of the current literature?

Yes

Reviewer Expertise:

Biochemistry,endocrinology,obstetrics and gynaecology,nutrition, pharmacology

I confirm that I have read this submission and believe that I have an appropriate level of expertise to confirm that it is of an acceptable scientific standard.

# Response

**Published:** 2024-01-20

**Authors:** Krishnananda Prabhu

**Affiliations:** Kasturba Medical College, Manipal, India

Dear Sir,

 Thank you very much for your valuable input to enrich this article. I shall do the required modification as soon as possible and resubmit for your approval.

 Regards,

# Reviewer report: Reviewer response for version 1

**DOI:** 10.5256/f1000research.137211.r210330

**Published:** 2023-10-10

**Authors:** Jufen Liu

**Affiliations:** 1 Peking University, Beijing, Beijing, China

The review is valuable and of great significance since multiple micronutrients were essential during pregnancy and the intricate relationship between micronutrients and adverse pregnancy outcomes were discussed in this review. For the lack of standardized micronutrient supplementation during pregnancy, the review could guide people to pay attention to optimizing the supplementation of relevant micronutrients and preventing hazards associated with its abuse. The review is clearly structured and easily understood. While the paper addresses a growing subpopulation of importance, the paper has some issues that could be addressed to improve the interpretations:

1. A brief introduction about the common micronutrients and related adverse pregnancy outcome should be given at the first beginning, which would be easily to retrieve the readers’ attention.
2. It mentioned that adverse pregnancy complications related to the overuse/unwanted use of multiple micronutrient supplementations during pregnancy in abstract as well as introduction, however, the article is more about micronutrient deficiencies. Consequently, some literature on over-supplementation of micronutrients could be added. And in the part of “Metabolic functions of micronutrients during pregnancy”, the status or relevant data of micronutrient supplementations could be mentioned to stress the significance of micronutrient supplementations.
3. In the introduction of various micronutrients in different pregnancy outcomes, the article seems to mainly focus on the role of micronutrients and is lack of research or experiments to prove its point forcefully. This leads to people not being aware of the importance of micronutrient supplementation. And the article shows that there are no proper validation/guidelines available concerning dosing/time, the need for initiation and duration of supplementation, etc. Perhaps some current research in this area could be added to arouse people's thinking.
4. It seems that the conclusion focuses more on the specific factors related mechanism, the public health significance of appropriate micronutrient supplementation during pregnancy should be added in conclusion.
5. The abstract and conclusion needs to be re-organized and revised.

Is the review written in accessible language?

Yes

Are all factual statements correct and adequately supported by citations?

Yes

Are the conclusions drawn appropriate in the context of the current research literature?

Partly

Is the topic of the review discussed comprehensively in the context of the current literature?

Yes

Reviewer Expertise:

Prenatal nutrition epidemiology

I confirm that I have read this submission and believe that I have an appropriate level of expertise to confirm that it is of an acceptable scientific standard, however I have significant reservations, as outlined above.

# Response

**Published:** 2023-10-10

**Authors:** Krishnananda Prabhu

**Affiliations:** Kasturba Medical College, Manipal, India

Dear Sir,

 Thank you very much for your valuable inputs. I shall accordingly include these suggestions and revert soon. This will go a long way in enriching this review article 

 Regards,

# Response

**Published:** 2023-11-03

**Authors:** Krishnananda Prabhu

**Affiliations:** Kasturba Medical College, Manipal, India

Dear Sir,

 Based on your suggestions, I have edited the article. Herewith sending it today to the editorial team for further processing. I sincerely thank for your valuable inputs 

 Regards,
